# Assessment of Overall Muscle Strength in Children and Adolescents Using Handheld Dynamometry: A Systematic Review of Reference Values and Quality of Data

**DOI:** 10.3390/jcm14238454

**Published:** 2025-11-28

**Authors:** Eleni Karagianni, Varsamo Antoniou, Zacharias Dimitriadis, Demosthenes Panagiotakos, Rita Cordovil, Garyfallia Pepera

**Affiliations:** 1 Clinical Exercise Physiology and Rehabilitation Research Laboratory (CeprLab) of Physiotherapy Department, University of Thessaly, 35132 Lamia, Greece; elkaragiann@uth.gr (E.K.); varsamoantoniou@uth.gr (V.A.); 2 Health Assessment and Quality of Life Lab of Physiotherapy Department, University of Thessaly, 35132 Lamia, Greece; zdimitriadis@uth.gr; 3 Department of Nutrition and Dietetics, School of Health Science & Education, Harokopio University, 17671 Athens, Greece; dbpanag@hua.gr; 4 Centro Interdisciplinar de Performance Humana (CIPER), Faculdade de Motricidade Humana, Universidade de Lisboa, Cruz Quebrada-Dafundo, 1499-002 Lisboa, Portugal; ritacordovil@fmh.ulisboa.pt

**Keywords:** children, adolescents, muscle strength, handheld dynamometry

## Abstract

**Background/Objectives:** Muscle strength is a vital indicator of the development and health-related fitness in children and adolescents, and it also plays a crucial role in clinical assessments across pediatric rehabilitation. The aim of this review was to evaluate the available literature on handheld dynamometry (HHD) assessments of the overall muscle strength in the pediatric population, focusing on measurement protocols, validity and reliability, and the availability of reference and normative data. **Methods**: Following PRISMA 2020 guidelines, a systematic review was conducted across PubMed, Scopus, Cochrane CENTRAL, CINAHL, and Web of Science. The protocol was registered in PROSPERO (CRD42024537557). Eligible studies (2005–2023) included healthy participants under the age of 18 reporting on isometric muscle strength using HHDs. Studies reporting handgrip strength or alternative methods (e.g., isokinetic and 1RM) of assessment were excluded. Due to the heterogeneity of the studies, the JBI and COSMIN risk of bias checklists were used to assess risk of bias. A narrative synthesis approach was used to summarize the findings. **Results**: Twelve studies were included, with a total of 1683 participants aged 4–17 years (764 girls and 919 boys). Test re-test reliability and low measurement error were established in measuring the toe strength, while the concurrent validity of lower limb torque was determined as poor. **Conclusions**: When standardized protocols and fixed stabilization are implemented, studies confirm good intra- and inter-rater reliability. Substantial heterogeneity in measurement protocols highlights the need for standardized procedures. It is essential to establish normative or reference values by age and sex to enhance clinical decision making and the utility of HHDs in pediatric overall strength assessments.

## 1. Introduction

Muscle strength, defined as the maximum force generated by a muscle or group of muscles, constitutes a pivotal element of health-related physical fitness in youth [1]. Evidence shows that children and adolescents with reduced overall muscle strength are at an increased risk of developing health issues [2,3]. Assessment of muscle strength plays a fundamental role in monitoring growth, physical development, and functional performance in children and adolescents. Accurate quantification of muscle strength is essential for clinical evaluation, rehabilitation planning, and sports participation monitoring, particularly for conditions such as neuromuscular disorders, obesity, and sedentary behavior [2,4,5].

Traditionally, clinicians and physical therapists have assessed muscle strength through manual muscle testing [6]. However, this approach is subjective and limited in accuracy. Isometric strength testing utilizing handheld dynamometers (HHDs) provides a more accurate and reliable alternative to manual muscle testing [7]. HHDs have become widely used in both research and clinical practice because they are portable, cost-effective, and feasible for use in diverse populations, including children [8,9]. Compared with isokinetic dynamometry—the gold standard—HHDs are more practical, although less standardized, as isokinetic devices require specialized equipment, space, and expertise [10,11].

Meanwhile, handgrip strength (HGS) has been the subject of extensive assessment and investigation in scientific literature. It is proposed to constitute a viable biomarker of healthy aging and a powerful predictor of future morbidity from youth to elderly populations [12,13,14]. Whilst the HGS dynamometer is a feasible, reliable, and highly accessible tool, it is often reported that there is a lack of comprehensive analysis when muscle mass or power data from the lower extremity or other muscle groups are not collected [15].

Muscle specific data are required in clinical evaluation and intervention strategies, in children and adolescents in sports specification, in various disorders, or in situations such as sedentary behavior and obesity [16]. Normative data obtained from typically developing children can be used as references from physiotherapists to detect muscle weaknesses or to determine the progress and the effectiveness of a chosen intervention. Two related, but distinct, terms are often used in the literature: normative data and reference values. Normative data describe the standard performance derived from large, representative populations, typically stratified by age and sex. Reference values, on the other hand, are obtained from well-defined samples under controlled conditions and allow for the interpretation of individual test results in clinical practice [17,18]. Despite their importance, existing studies often use these terms interchangeably, leading to ambiguity, and the lack of clarity and uniformity in applying these definitions contributes to further challenges in synthesizing existing evidence.

To our knowledge, no systematic review has comprehensively summarized the evidence on the use of HHDs in children and adolescents. Therefore, the objective of this review was to evaluate the available literature on HHD assessments of muscle strength in the pediatric population, focusing on measurement protocols, validity and reliability, and the availability of reference and normative data. Establishing standardized methods and values is essential for identifying strength impairments and guiding interventions in clinical and sports settings.

## 2. Materials and Methods

### 2.1. Study Design

This systematic review was conducted in accordance with the Preferred Reporting Items for Systematic Review (PRISMA 2020) guidelines (Supplementary File S1) [19]. The protocol was prospectively registered in PROSPERO (International Prospective Register of Systematic Reviews) (registration number: CRD42024537557).

### 2.2. Study Selection Process

Following the exclusion of duplicates, two reviewers (EK and GP) independently screened the titles and abstracts of the studies in the finalized search list. The reviewers conducted full-text screening of the remaining relevant papers to determine their eligibility according to the review criteria. Articles that failed to meet the eligibility criteria were excluded.

### 2.3. Study Eligibility

#### Inclusion Criteria

In our review, we included observational and interventional studies that were published in English in peer-reviewed journals. Studies were eligible for inclusion in the review if the data (or part of the presented data) concerned muscle strength being measured using HHDs in children and adolescents (under 18 years old).

### 2.4. Exclusion Criteria

Studies were excluded if they were published in a language other than English, or if the mean age of the participants was greater than 17.99 years. Studies that measured HGS, as well as those that implemented alternative methods of measuring strength (such as the one repetition maximum (1RM) method, functional tests, or isokinetic dynamometers), were excluded. Studies on clinical populations with specific disorders were also excluded, unless data for healthy participants were separately reported.

### 2.5. Search Strategy

A comprehensive electronic literature search was conducted across five electronic databases to identify pertinent studies: PubMed, Scopus, Cochrane CENTRAL, CINAHL, and Web of Science (Supplementary File S2). The studies relevant for inclusion were searched from January 2005 up to December 2023 to ensure the most up-to-date evidence. Each database search utilized the same keywords to identify the specific field of interest: ‘children’ OR ‘adolescents’ AND ‘handheld’ OR ‘dynamometry’ OR ‘muscle strength’ OR ‘data’. The terms ‘students’, youth’, schoolchildren’, ‘maximum voluntary muscle contraction’, and ‘muscle strength values’ were also used as specific key words. Only articles that were relevant and written in English were included in the final list of the systematic review. Papers that met the search criteria were exported to Mendeley Reference Manager for reference management. Duplicate records were initially identified and removed automatically by the software based on matching titles, authors, and publication details. A subsequent manual review was then performed to ensure that any remaining duplicates were eliminated before proceeding to the screening phase.

### 2.6. Data Extraction

Data extraction (Table 1) was conducted on the selected studies, encompassing the following domains: (1) study design and setting (first author, year of publication, and country); (2) participants and characteristics (sample size, sex, and age); (3) testing procedures and device characteristics (type of instrument, best effort recording, mode of contraction, stabilization method, and number of trials); (4) muscle groups assessed and testing positions; and (5) outcomes reported (reliability, validity, and reference/normative values).

### 2.7. Risk of Bias (Quality) Assessment

Given the heterogeneity of study designs, three different tools were used to evaluate the risk of bias assessment. (Table 2, Table 3 and Table 4) Specifically, the Joanna Briggs Institute Critical Appraisal tools [20] in the JBI Systematic Checklist for Analytical Cross Sectional Studies was used to determine the methodological consistency of eight of the selected articles. The JBI critical appraisal checklist for cohort studies was used for one of the studies included, and the COSMIN risk of bias [21] checklist (Boxes 6 and 8) was used for three of the studies in this systematic review. The purpose of using the JBI checklist for Analytical Cross Sectional Studies was to evaluate the methodological quality of a study and to ascertain the presence of any potential bias in its design, execution, and analysis. It consists of a total of eight items, where reviewers assess compliance with the checklist with the following answers: YES, NO, UNCLEAR, and NOT APPLICABLE. Similarly, the JBI checklist for cohort studies consists of eleven items that help examine the selection bias, exposure measurement, outcome measurement, confounding factors, follow-up, and statistical analysis. The COSMIN risk of bias tool assesses reliability and validity. The overall score is determined by taking the lowest rating of any standard in the box. Two independent reviewers (EK and PG) conducted the quality appraisal individually. More than half of the stipulated checklist items were met. Any discrepancies were resolved via consultation with a third reviewer (VA). In instances where studies failed to meet the majority of the stipulated checklist items or where the information provided lacked the necessary specificity, they were excluded from the study.

### 2.8. Data Synthesis and Grouping

Documentation of the data from each study, with the objective of collating information regarding the variables of the data extraction grid (Table 1), was conducted using Microsoft Excel spreadsheets. Data extraction and management from the included studies were conducted in accordance with the PRISMA recommendations 2020 expanded checklist [19]. The following information was extracted from each study: authors; year of publication; country or region; number of participants; sex; age (or reported age groups per year); type of dynamometer and force recorded; testing procedures; type of muscle contraction and sides tested; muscle groups evaluated and assessment position; and—finally—each study’s objective.

To synthesize the findings, studies were grouped primarily by the muscle groups assessed (upper limb, lower limb, or both) and secondarily based on the stabilization technique applied (fixed versus tester-involved dynamometry). This approach allowed for a structured comparison of measurement protocols and outcomes across studies.

## 3. Results

### 3.1. Study Selection

**Figure 1:**
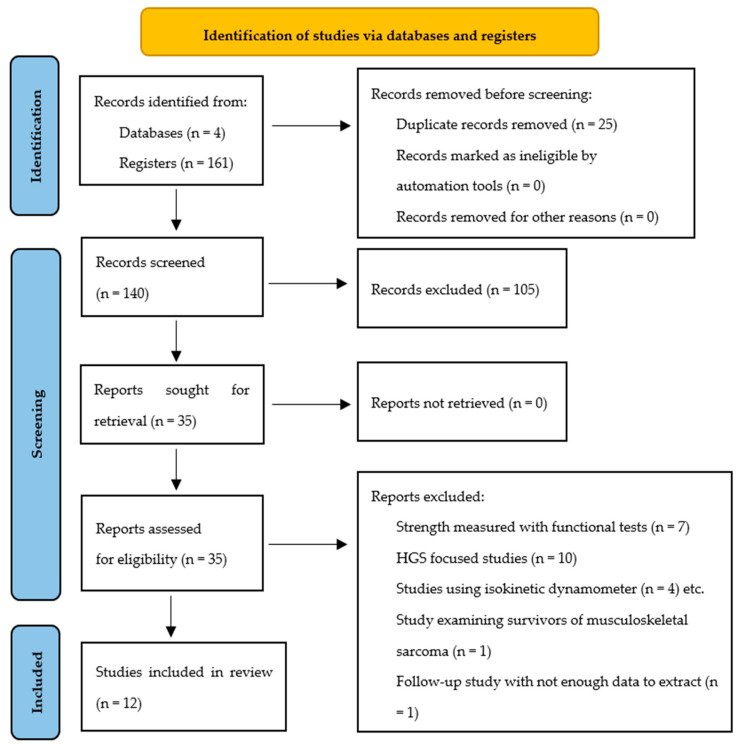
PRISMA flow diagram.

Figure 1 presents the study selection process. A total of 165 records were identified from the different databases, and 25 duplicates were removed. Of the 140 titles and abstracts screened, 105 were excluded due to ineligible content. Finally, 35 reports were identified for full-text retrieval and then screened for eligibility criteria. A total of twenty-three (23) full-text articles were excluded with justification (reasons included: use of functional strength tests [*n* = 7], HGS-only assessments [*n* = 10], isokinetic dynamometry [*n* = 4], and non-extractable data [*n* = 2]). Ultimately, 12 articles met the inclusion criteria and were included in this review (Table 5).

### 3.2. Study Characteristics

The included studies comprised a total of 1683 participants aged 4–17 years (764 girls and 919 boys). Sample sizes ranged from 14-to-400 participants. Two studies included only male participants [25,26].

We identified six different HHD devices used to assess muscle strength: MicroFET2 (Hoggan Scientific, LLC, Salt Lake City, UT, USA); Lafayette Instrument Models 01163 and 01165 (Lafayette Instrument Company, Lafayette, IN, USA); FCE-500 Ametek TCI Division (C.S.C. Force Measurement, Inc., Agawam, MA, USA); GS Gatherer and GS Analysis Suite^®^ (Gatherer Systems Limited, Aylesbury, UK); Wagner ForceOne FDIX (Wagner Instruments, Greenwich, CT, USA) ; and JTECH Commander PowerTrack Muscle Dynamometer (JTECH Medical, Midvale, UT, USA). Reported outcomes included the maximal isometric force or torque, expressed in Newtons (N), Newton-meters (Nm), kilograms (kg), or pounds (lb). Some studies normalized force values to body mass, while others reported the raw force or torque values.

Of the 12 studies included, 4 used external stabilization with fixations on the dynamometer, so the evaluator was not physically involved in the application of force. One study [30] used stabilization over the contralateral leg and torso if needed. One of the four studies [5], however, used stabilization of the dynamometer for the muscle groups of hip flexors and extensors and not for the rest of the muscle groups. More specifically, the examiner used a nonelastic strap to create resistance in a pulling direction. This prevented the nonisometric muscle contraction that may be caused by particularly strong muscle groups. The mode of the HHDs was set to compression in most of the studies, and only Hebert et al. (2015) [5] disclosed on using the distraction as referred to above. In one of the studies, the testing figure suggested that the distraction mode was most likely used, as the strap was shown secured to a wall surface while the participant, positioned prone on a treatment table, exerted force by pulling against it during knee flexion [25]. Similar to the study of Šarčević et al. (2020) [27], the mode was assumed to be compression from the photos reported. All 12 studies had one-to-three examiners/evaluators except for one study, which had six in total (two for each school included), and another study (whose exact number of examiners was not reported).

### 3.3. Risk of Bias Assessment

Most cross-sectional studies scored positively on the JBI checklist, with occasional unclear reporting regarding confounding factors and statistical methods. The cohort study [23] was judged to be of moderate quality, mainly due to the limited reporting for follow-ups. Additionally, the three studies assessed with the COSMIN reliability tool [28,29,30] demonstrated adequate-to-very-good methodological quality.

### 3.4. Muscle Groups

There was variability in the studies included regarding the muscle group that was tested. One study focused solely on identifying the muscle strength profile of upper limbs in children and adolescents [7]. Five studies examined lower limb strength, specifically targeting the knee flexors and extensors [25,26], the knee extensors from different testing positions (seated and supine) [28], the toe plantar flexors [29], and total lower limb strength [30] (including hip flexion, adduction (ADD), abduction (ABD), knee flexion and extension, and ankle plantar flexion and dorsiflexion). The following six of the twelve studies evaluated both the upper and lower limb strength, including ABD, ADD flexors and extensors of the hip, and flexors and extensors of the elbow and wrist, as well as ankle plantar flexors and dorsiflexors [4,5,22,23,24,27]. In the study conducted by Šarčević et al. (2020) [27] the lower trapezius, serratus anterior, and erector spinae muscles were included in the analysis.

### 3.5. Summary of Evidence on Measurement Properties

This systematic review included a cohort study [23], where the concurrent validity of the HHD protocol with the Cybex dynamometer was verified. In the same study, the maximal isometric torque values demonstrated acceptable intra- and inter-rater reliability, whereas the ankle dorsiflexors showed poor intra- and inter-rater reliability. Test–retest reliability and a low measurement error were also established [29] [hallux [ICC (3,3) = 0.93, 95% CI 0.78–0.98, SEM = 4.31 N]. In addition, the lesser toe flexor strength [ICC (3,3) = 0.96, 95% CI 0.87–0.99, SEM = 1.86 N] in measuring the toe strength in children using a novel fixed dynamometer [29] and Rock et al. (2021) [28] suggest that HHDs are a reliable tool (TEM = 2.38 Nm, CV% = 7.07–994% for all three trials and TEM = 161 Nm, CV% = 4.49–5.61% for the two best efforts) for measuring quadriceps strength, especially when two of the best out of the three attempts are used in the final analysis. The concurrent validity of lower limb torque using an HHD was determined as poor against the gold standard measure of isokinetic dynamometry in children [30].

Two correlation studies were included, where one investigated the effects of body mass on the peak torque in schoolchildren [26] and the other identified correlations between the maximum isometric strength and the inclination angles of the curvatures of the spine [27]. One study [25] reported the IVMCmax of quadriceps and hamstrings of youth football players, as well as the isometric hamstring/quadriceps ratio and muscle strength asymmetries between limbs.

Four studies explicitly provided reference or normative strength values for specific age groups [4,5,7,24]. However, definitions of “normative” versus “reference” values were not consistently reported.

## 4. Discussion

Despite the growing use of HHDs in pediatric populations, the literature remains inconsistent, with considerable variability in testing protocols, measurement units, and reporting standards. Moreover, normative and reference data for children and adolescents are limited and inconsistently defined, making clinical interpretation and comparison across studies challenging. Addressing these gaps is essential for establishing standardized assessment methods and improving the reliability of strength evaluation in pediatric clinical and sports practice.

The aim of this systematic review was to identify all the available data on the use of HHDs in children and adolescents. Following a thorough review of the available literature, only 12 studies met the inclusion criteria. The findings indicate that HHDs are a feasible and reliable tool for pediatric populations. Several studies confirmed good intra- and interrater reliability, particularly when standardized protocols and fixed stabilization were applied. Reference or normative values were reported in a subset of studies, but definitions and methodologies varied widely, preventing the establishment of universal normative datasets.

Due to the heterogeneity of the methodology followed by the studies, there were no sufficient data for normative data to be extracted. The titles of the extracted articles varied, and it is noteworthy that none of the studies provided definitions for the terms “reference values” or “normative data’’. In four of the studies identified, the terms ‘torque reference values’ [5], ‘normative data’ [4], ’isometric strength profile’ [7], and ‘strength values’ were found [24]. A study published in 2001 [31], using the term ‘reference values of muscle force’, was not included in the review since the inclusion criteria required a specific time frame. There is a distinct difference in the terms ‘reference values’ and ‘normative data’ that are used in the literature [18]. Normative values refer to values ‘relating to or determining norms or standards’, and they are defined as ‘a set standard of development or achievement, usually delivered from the average or median achievement of a large group’ [32]. A reference value is defined as the values obtained from individuals presenting similar conditions with the sample tested in a well-controlled environment, allowing for adequate interpretation of the test results [17].

There is limited research on the use of manual dynamometry in the pediatric population, with significant gaps remaining in the existing literature [33,34]. It is acknowledged that there is a lack of methodology protocols and a variety of study designs regarding the devices used for HHDs, the mode of contraction, the positioning, the assessment methods, etc. A scoping review [18] investigated the use of HHDs in adults, and it was noted that different devices were used with no validity between the tools. Also, due to the different force capacities of the devices, a ceiling effect would be created when testing muscle groups (e.g., knee extensors) that exceeded the devices’ upper force limit. We consider this a limitation that was not addressed in the included studies, particularly for children—especially older adolescents—who exhibit greater muscle force.

More than half of our studies reported strength values in units of torque (Nm) or force (N) divided by the body mass of each participant. We acknowledge a paucity of literature on this specific subject, since studies in the existing literature did not consider the anthropometric characteristics of the participants, which can greatly influence the torque that can be generated. In the study by Mendez-Rebolledo et al. (2022) [7], the isometric muscle strength of the upper limbs of children was normalized by body mass. Participants were categorized into yearly age ranges, which permitted the assumption that there was no statistical difference in anthropometric data within a specific age range. This could potentially ensure that muscle strength data would be representative of a given age and sex [35]. Another study [36] measured the hip strength of women and presented absolute values expressed as a percentage of body weight, which facilitates the clinical use of dynamometry. This could enable professionals to assess the loss of muscle strength; thus, normalizing values could be obtained without the need of regression calculations [37,38]. An important parameter that takes individuals’ body segment length differences into account is the length of the lever arm (i.e., the perpendicular distance between the placement of the HHD and the axis of rotation of the tested segment) [18]. It is highlighted that the so-called raw HHD output of muscle force may be misleading as the evaluation depends on the distance between the dynamometer and the tested joint [35]. When examining children, we ought to consider their growth [39], where limb length influences the distance of the joint center and torque calculation is considered necessary [40]. Identifying that there was a lack of torque measurement while using an HHD in four of the studies included in this systematic review highlights the possible misconception of the use of muscle force data and values in clinical practice.

Despite studies in the literature and those included in this systematic review reporting that examiners receive standardized training regarding the usage of HHD, this type of methodology adds bias to the procedure [5,18,41]. When examining muscle strength and there is insufficient stabilization, compensatory movements may affect the peak force generation by a person. Large muscle groups (e.g., knee and hip muscles) may produce forces the examiner will not be able to resist [42]. We also acknowledged that the studies included used different positioning methods that could not be reproduced using a standardized approach. Differences in the use of stabilization through an inelastic belt may not be present in younger ages due to their lower torque values. External stabilization has an advantage when higher torque values are achieved, which depends on the age, height, weight, or physical performance of children and adolescents.

Furthermore, given the younger age groups, misinterpretations of the procedures are to be expected in manual dynamometry or dynamometry in general. It is, therefore, imperative to standardize the protocols in use regarding the following: the verbal instructions and encouragement used before and during testing; the positioning of both examiner and the participant; the position of the HHD and possible accessories; the order of the muscles tested; and the execution of the task by the participant [23]. Authors should discuss the results and how they can be interpreted from the perspective of previous studies and working hypotheses. The findings and their implications should be discussed in the broadest context possible. Future research directions may also be highlighted.

Overall, the confidence in the body of evidence synthesized in this review is moderate to low. According to the GRADE assessment, the certainty of evidence for the main outcomes ranged from low to very low; details are provided in Appendix A. Several factors limit the strength and generalizability of the conclusions. The included studies demonstrated considerable heterogeneity in testing protocols, stabilization methods, and outcome measures, which complicated cross study comparisons. Most studies were based on small, convenient samples, often lacking adequate representation across age, sex, and activity levels. Furthermore, the absence of meta-analytic synthesis restricted the ability to quantify pooled reliability or validity estimates. Collectively, these factors reduce the certainty of the evidence and caution against broad generalization of the findings to all pediatric populations or clinical contexts.

One of our study’s limitations was the language restriction to review only English-written literature, which may have influenced the number of the studies included in this systematic review and could have potentially resulted in selection bias. However, the restriction was applied primarily to ensure accurate data extraction, interpretation, and methodological consistency, as translation of non-English manuscripts could have introduced linguistic and contextual inaccuracies. Additionally, the majority of the high-quality, peer-reviewed research in this field is published in English, minimizing the potential impact of language bias.

Despite methodological variability, HHDs remain a valuable tool in pediatric clinical and sports settings, offering an objective and portable means of assessing muscle strength across a wide age range. The data obtained can support rehabilitation planning, injury prevention strategies, and the monitoring of growth-related changes in strength. However, the absence of standardized protocols and normative datasets continues to limit the interpretability and comparability of results across studies and populations.

Future research should aim to strengthen scientific rigor by establishing standardized testing protocols that clearly define instructions, participant positioning, number of trials, and stabilization methods. Studies should include large and representative pediatric cohorts and adopt torque-based reporting that incorporates lever arm length to account for differences in growth and body size. Additionally, regular device calibration and capacity testing are essential to avoid ceiling effects, particularly in older adolescents. Finally, future investigations should explore the clinical applicability of HHDs in specific pediatric populations, such as those with obesity, sedentary lifestyles, or varying levels of sports participation.

## 5. Conclusions

HHDs are a valuable tool in clinical practice that can be used in various areas of rehabilitation, such as injury prevention, musculoskeletal impairment, spine health, and cardiovascular health [27,43,44]. The evidence synthesized in this review indicates that HHDs can be successfully applied across different muscle groups and age ranges, and several studies have established preliminary reference values. However, methodological heterogeneity, including variability in protocols, outcome measures, and definitions of reference versus normative data, limits the comparability of findings and prevents the establishment of robust normative datasets. Future research should focus on the development of standardized assessment protocols, incorporation of anthropometric adjustments such as lever arm length, and the recruitment of large, representative samples. These steps are essential to generate clinically meaningful normative values that can guide rehabilitation, sports training, and health monitoring in the pediatric population.

## Figures and Tables

**Table 1:** Data extraction grid.

Data Extracted
Authors Year Country Sample Age Sex Number of participants HHD model Measurement units HHD maximal capacity (N, lb, kg) Mode (compression/traction) Contraction type Instructions Protocol reproducibility (positioning for measurement) HHD placement Muscle groups tested Results Limits reported Other

**Table 2:** The risk of bias, as per the JBI Critical appraisal for Cohort Studies.

JBI Cohort	1	2	3	4	5	6	7	8	9	10	11
Hebert et al., 2011 [22]	Yes	Yes	Yes	Yes	Unclear	No	Yes	Yes	Yes	Yes	Yes

**Table 3:** The risk of bias, as per the JBI Critical appraisal for Cross Sectional Studies.

JBI Cross Sectional	1	2	3	4	5	6	7	8
Macfarlane et al., 2008 [23]	Yes	Yes	Yes	Yes	Yes	No	Yes	Yes
Hebert et al., 2015 [5]	Yes	Yes	Yes	Yes	Yes	Unclear	Yes	Yes
Escobar et al., 2017 [24]	Yes	Yes	Yes	Yes	Yes	Yes	Yes	Yes
Daloia et al., 2018 [4]	Yes	Yes	Yes	Yes	Unclear	Unclear	Yes	Yes
Peek et al., 2018 [25]	Yes	Yes	Yes	Yes	Unclear	Unclear	Yes	Yes
Alhusaini et al., 2019 [26]	Yes	Yes	Yes	Yes	Unclear	Unclear	Yes	Yes
Šarčević et al., 2019 [27]	Yes	Yes	Yes	No	Yes	Unclear	Yes	Yes
Mendez-Rebolledo et al., 2022 [7]	Yes	Yes	Yes	Yes	Yes	Unclear	Yes	Yes

**Table 4:** The risk of bias, as per the COSMIN risk of bias tool.

COSMIN Risk of Bias	Were Patients Stable in the Interim Period on the Construct to be Measured?		Was the Time Interval Appropriate?		Were the Test Conditions Similar for the Measurements? E.g., Type of Administration, Environment, Instructions	For Continuous Scores: Was an Intraclass Correlation Coefficient (ICC) Calculated?		Were There Any Other Important Flaws in the Design or Statistical Methods of the Study?		Overall Methodological Quality
Box 6 Reliability										
Rock et al., 2021 [28]	Adequate		Very good		Very good	Very good		Very good		Adequate
Quinlan et al., 2021 [29]	Very good		Very good		Very good	Very good		Very Good		Very good
**Box 8 Criterion Validity**		**Statistical methods**		**Other**			**Overall methodological quality**			
Mahafey et al., 2022 [30]		Very good		Very good			Very good			

**Table 5:** Study characteristics.

Authors	Country/Region	Participants	Age (Mean ± SD)	Type of Dynamometer and Measurement Unit	Testing Procedures	Type of Muscle Contraction and Sides Tested	Muscle Groups and Assessment Position	Study Objective
Macfarlane et al., 2008 [23]	Michigan, USA	No = 154 F = 90 M = 64	Pooled for age, 6–8 age groups	MicroFET2 [Hoggan Scientific, LLC, Salt Lake City, UT, USA] Force: N	Mode: Compression Test trials: 3 Total test duration: 25–30 min Evaluators: 1 Stabilization: No	Contraction type: Make test Isometric [Bilateral]	Hip ABD/ADD: Supine Hip flexors/extensors: Sidelying [Hips and knees flexed at 45°] Knee flexors/extensors: Seated	Establishing muscle force and torque reference values for 6-to-8 years old
Hebert et al., 2011 [22]	Québec, Canada	No = 74 F = 37 M = 37	10,7 ± 4 [4–17.5 years old 9 age groups]	[1] Lafayette Instrument, Model 01163 [Lafayette Instrument Company, Lafayette, IN, USA] and [2] FCE-500 Ametek TCI Division [C.S.C. Force Measurement, Inc., Agawam, MA, USA] Force: N, Torque: Nm	Mode: Compression and Distraction for lower limb muscles Test trials: 2 Total test duration: 45 min, 4 sessions, 5–14 days apart Evaluators: 2 Stabilization: Yes for knee extensors, No for the rest of muscle groups	Contraction type: Make test Isometric [Bilateral for older participants and randomly selected one sided test for ages 4–8.5]	Shoulder ABD: Supine [1] Shoulder LR: Supine [1] Elbow flexors/extensors: Supine [1] Hip flexors/extensors: Supine [2] Hip ABD: Supine [1] Knee flexors/extensors: Seated [1] Ankle plantar flexors/dorsiflexors: Supine [1]	Feasibility, intra- and interrater reliability, SEMs, concurrent validity statistics
Hebert et al., 2015 [5]	North America	No = 348 F = 174 M = 174	Pooled for age, 4–17 age groups	FCE-500 Ametek TCI Division [C.S.C. Force Measurement, Inc., Agawam, MA, USA] Torque: Nm	Mode: Compression and Distraction for hip flexors and extensors only Test trials: 3 Total test duration: Not disclosed Evaluator: 6 (2 Per school) Stabilization: Yes, for hip flexors and extensors, not for the rest of muscle groups	Contraction type: Make test Isometric [Bilateral for older participants and randomly selected one sided test for ages 4–8.5]	Shoulder ABD and ER: Supine Elbow flexors/extensors: Supine Hip flexors/extensors: Supine Hip ABD: Supine Knee flexors/extensors: Seated Ankle dorsiflexors: Supine	Reference values establishment Comparison of torque between absolute and reference values Comparison of muscle strength profiles
Escobar et al., 2017 [24]	Chile	No = 400 F = 213 M = 187	Pooled for age, 6–15 age groups	Lafayette Instrument, Model 01163 [Lafayette Instrument Company, Lafayette, IN, USA] Force: N	Mode: Compression Test trials: 3–5 Total test duration: 30–40 min Evaluators: 3 Stabilization: No	Contraction type: Break test Isometric [Bilateral]	Shoulder flexors/ABD: Seated Elbow flexors/extensors: Supine Wrist flexors/extensors: Supine Hip flexors/ABD: Supine Knee extensors: Seated Ankle plantar flexors/dorsiflexors: Supine	Muscle strength values determination
Daiola et al., 2018 [4]	São Paulo, Brazil	No = 110 F = 55 M = 55	Pooled for age, 5–15 age groups	Lafayette Instrument [Lafayette Instrument Company, Lafayette, IN, USA] Force: N	Mode: Compression Test trials: 3 Total test duration: Not disclosed Evaluators: 2 Stabilization: No	Contraction type: Not disclosed [Push command] Isometric [Bilateral]	Shoulder ABD: Supine Elbow flexors/extensors: Supine Knee flexors/extensors: Seated Ankle plantar flexors/dorsiflexors: Supine	Description of the development of isometric muscle strength and the evaluation of inter and intra-rater reliability
Peek et al., 2018 [25]	Australia	No = 142 M = 142	Pooled for age, 8–15 age groups	GS Gatherer and GS Analysis Suite^®^ [Gatherer Systems Limited, Aylesbury, UK] Force: N	Mode: Not disclosed [Assumed Distraction] Test trials: 2 Total test Duration: 20–30 min Evaluators: 1 Stabilization: Yes	Contraction type: Break test Isometric [Bilateral]	Knee flexors/extensors: Prone [Hamstring/quadriceps ratio]	Investigation of IVMCmax characteristics of the hamstrings and quadriceps in youth football players
Alhusaini et al., 2019 [26]	Riyadh, Saudi Arabia	No = 51 M = 51	14.17 ± 0.71	Lafayette Instrument, Model 01165 [Lafayette Instrument Company, Lafayette, IN, USA] Force: N	Mode: Compression Test trials: 3 Total test duration: Not disclosed Evaluators: Not disclosed Stabilization: No	Contraction type: Make test Isometric [Bilateral]	Knee flexors/extensors: Seated	Relationship between BMI scores, LQYBT performance, and the lower-limb strength of schoolchildren
Šarčević et al., 2019 [27]	Novi Sad, Serbia	Νo = 63 F = 32 M = 31	12.73 ± 1.58	JTECH Commander PowerTrack Muscle Dynamometer [JTECH Medical, Midvale, UT, USA] Force: N	Mode: Assumed Compression Test trials: 3 * Total test duration: Not disclosed Evaluators: 1 Stabilization: Yes	Contraction mode: Not disclosed Isometric [there was no side-testing disclosure.]	DMN Lower trapezius: Prone row position. DMN Serratus anterior and upper trapezius: Seated 120–130° Shoulder flexion Hip ABD: Supine Hip extension: Prone with the knee flexed to 90° Erector spinae: on hands and knees bent at 90°	Correlation study between maximum isometric strength in five muscle groups
Rock et al., 2021 [28]	Baltimore, MD, USA	No = 23 F = 13 M = 10	7.18 ± 1.15 [6–8 years old] 10.16 ± 0.71 [9–11 years old] 14.74 ± 2.01 [12–17 years old]	Lafayette Instrument, Model 01165 [Lafayette Instrument Company, Lafayette, IN, USA] Force: N	Mode: Compression Test trials: 3 Total test duration: Not disclosed Evaluators: 1 Stabilization: No	Contraction type: Not disclosed Isometric [Dominant lower extremity]	Knee extensors: seated 90° and 35°; supine 90° and 35°	Reliability of the use of HHDs and ultrasonography to measure quadriceps strength
Quinlan et al., 2021 [29]	Australia	No = 14 F = 8 M = 6	11.25 ± 0.38	Lafayette Instrument, Model 01163 [Lafayette Instrument Company, Lafayette, IN, USA] Force: N	Mode: Compression Test trials: 2 Total test duration: Not disclosed [2 sessions, 7–14 days apart] Evaluators: 1 Stabilization: Yes	Contraction type: Not disclosed Isometric [Dominant foot]	Toe plantar flexors: seated [Hallux and Lesser toes]	Test–retest reliability of a novel fixed HHD protocol
Mahafey et al., 2022 [30]	United Kingdom	No = 61 F = 28 M = 33	9.20 ± 0.98	Wagner ForceOne FDIX [Wagner Instruments, Greenwich, CT, USA] Force: N Torque: Nm	Mode: Assumed Compression Test trials: 2 Total test duration: approx. 15 min Evaluators: 2 Stabilization: Yes, on the contralateral leg	Contraction type: Make test Isometric [dominant lower extremity]	Hip flexion: Supine Hip ADD/ABD: Sidelying Knee flexors/extensors: Seated Ankle plantar flexors/dorsiflexors: Prone	Concurrent validity of lower limb torque from HHDs compared to isokinetic dynamometry in children aged 7-to-11 years old
Mendez- Rebolledo et al., 2022 [7]	Chile	No = 243 F = 114 M = 129	Pooled for age, 7–15 age groups	Lafayette Instrument, Model 01165 [Lafayette Instrument Company, Lafayette, IN, USA] Force: N; N/Kg (Body mass)	Mode: Compression Test trials: 3 Total test duration: 60 min Evaluators: 1 Stabilization: No	Contraction type: Make test Isometric [Dominant upper extremity]	Shoulder ABD/flexors/LR /MR: Seated Elbow flexors/extensors: Supine Wrist flexors/extensors: Supine	Determining the isometric strength profile of the upper limb muscles of children and adolescents

* Authors refer to the three correct trials being used in data analysis. They do not disclose if any participants needed extra attempts to correctly complete the tests. Abbreviations are listed at the end of the manuscript.

## Data Availability

No new data were created or analyzed in this study.

## Supplementary Materials

The following supporting information can be downloaded at: https://www.mdpi.com/article/10.3390/jcm14238454/s1, Supplementary File S1: PRISMA 2020 Checklist; Supplementary File S2: Database search strategy.

## Abbreviations

The following abbreviations are used in this manuscript:

ABD	Abductor
ADD	Adductor
DMN	Dominant
ER	External rotation
F	Female
HGS	Handgrip strength
HHD	Handheld dynamometer
IVMC	Isometric voluntary muscle contraction
JBI	Joanna Briggs Institute
kg	Kilogram
Lb	Pound
LQYBT	Lower Quarter Y Balance Test
LR	Lateral rotation
M	Male
Min	Minutes
MIT	Maximal isometric torque
N	Newton
Nm	Newton-meter
No	Number of participants
MR	Medial rotation
PRISMA	Preferred Reporting Items for Systematic Reviews

## Appendix A

This table presents the GRADE assessment of the certainty of evidence regarding the associations between HHDs and overall muscle strength assessments in children and adolescents. The evaluation considered the study design, risk of bias, consistency, directness, and precision. The table provides a transparent summary of the strength and quality of the available evidence supporting the use of HHDs in pediatric populations.

**Table A1:** GRADE summary of the findings, assessing the certainty of evidence for associations between HHDs and overall muscle strength assessments in children and adolescents.

No of Studies	Study Design	Risk of Bias	Inconsistency	Indirectness	Imprecision	Other Considerations	Intervention	Comparison	Certainty Importance	
1 [22]	Non-randomized studies Cohort study	Not serious	Not serious	Not serious	Not serious	None	74/74 (100.0%)	37/74 (50.0%)	Low	Acceptable intra- and inter-rater reliability and concurrent validity with the Cybex.
8 [4,5,7,23,24,25,26,27]	Non-randomized studies Cross-sectional studies	Serious *	Not serious	Not serious	Not serious	None	−1511	1511/1511 (100.0%)	Very low	Variation among studies regarding the muscle groups tested, fixation protocols, and data analysis or presentation.
3 [28,29,30]	Non-randomized studies Reliability and Criterion Validity	Not serious	Not serious	Not serious	Not serious	None	98	98/98 (100.0%)	Low	Test re-test reliability and low measurement error for hallux and lesser toe flexor strength. Reliable knee extension joint torque measurements using HHDs. Poor concurrent validity of HHDs in children 7–11 years old compared to Isokinetic Dynamometer

* Most of the studies did not refer to any confounding factors that could influence the results, nor did they implement any strategies to address potential confounding factors likely to arise in HHD measurements.
