# Supplementary material for: Assessment of Overall Muscle Strength in Children and Adolescents Using Handheld Dynamometry: A Systematic Review of Reference Values and Quality of Data
Source: J Clin Med. 2025 Nov 28;14(23):8454. doi: 10.3390/jcm14238454 (PMC12693157; doi:10.3390/jcm14238454)
Supplement: Supplementary file 1 [file jcm-14-08454-s001.zip › jcm-3952760-supplementary-S2.pdf]

Database search strategy *Supplementary file 2*

Database	Search Strategy	Date Searched	Limits / Filters
**PubMed**	{"children" [MeSH Terms] OR "adolescent" [MeSH Terms] OR youth OR students OR "schoolchildren"} AND {"handheld" OR "hand-held" OR "dynamometry" OR "handheld dynamometer" OR "muscle strength" OR "maximum voluntary muscle contraction" OR "muscle strength values" OR "data" OR ''assessment''}	December 2023	English language; publication date from 2005 to 2023
**Scopus**	TITLE-ABS-KEY ("children" OR "adolescents" OR "youth" OR "students" OR "schoolchildren") AND TITLE-ABS-KEY ("handheld" OR "hand-held" OR "dynamometry" OR "handheld dynamometer" OR "muscle strength" OR "maximum voluntary muscle contraction" OR "muscle strength values" OR "data" OR ''assessment'')	December 2023	English; 2005–2023
**Cochrane CENTRAL (Cochrane Library)**	("children" OR "adolescents" OR "youth" OR "students" OR "schoolchildren") AND ("handheld" OR "hand-held" OR "dynamometry" OR "handheld dynamometer" OR "muscle strength" OR "maximum voluntary muscle contraction" OR "muscle strength values" OR "data" OR ''assessment'')	December 2023	2005–2023; English
**CINAHL (EBSCOhost)**	("Children" OR "Adolescence" OR ''children'' OR ''adolescents'' OR ''youth'' OR ''students'' OR "schoolchildren") AND (''handheld'' OR "hand-held" OR ''dynamometry'' OR "handheld dynamometer" OR "muscle strength" OR "maximum voluntary muscle contraction" OR "muscle strength values" OR ''data'' OR ''assessment'')	December 2023	English; Published 2005–2023
**Web of Science**	("children" OR "adolescents" OR "youth" OR "students" OR "schoolchildren") AND ("handheld" OR "hand-held" OR "dynamometry" OR "handheld dynamometer" OR "muscle strength" OR "maximum voluntary muscle contraction" OR "muscle strength values" OR "data" OR ''assessment'')	December 2023	English; 2005–2023
